# Integrative Nutrition CARE in the Community—Starting with Pharmacists

**DOI:** 10.3390/pharmacy8030170

**Published:** 2020-09-13

**Authors:** Chun-Wai Mai, Jennifer See Hui Tan, Gina Wan Lee Koay, Lucas Yang Xian Lim

**Affiliations:** 1 Department of Pharmaceutical Chemistry, School of Pharmacy, International Medical University, No.126, Jalan Jalil Perkasa 19, Bukit Jalil, Kuala Lumpur 57000, Malaysia; 2 Centre for Cancer and Stem Cells Research, Institute for Research, Development and Innovation, International Medical University, Kuala Lumpur 57000, Malaysia; 3 E&G Scientific Sdn Bhd, Selangor 48000, Malaysia; jennifertanseehui@gmail.com; 4 City Wellness Pharmacy, 1-G, Lorong Delima 5, Island Glades, Penang 11700, Malaysia; ginakoay1@gmail.com; 5 Dietitian90 Consultancy, 99-1-8 Bazaar Tanjung, Jalan Fettes, Tanjung Bungah, Penang 11200, Malaysia; lucaslimyangxian@gmail.com

**Keywords:** community pharmacists, supplementation, consultation, health promotion, dietetics

## Abstract

Dietary supplementation is increasingly sought after by consumers looking to meet the demands of a modern lifestyle. Effective supplementation requires knowledge of the purpose and proper use of nutritional supplements. Unverified or inadequate guidance on supplementation can propagate misconceptions and increase undue fears of side effects. Community pharmacists are best placed to guide consumers on nutritional supplement use. In this review, a panel comprised of community pharmacists, pharmacy academia, and dietitians (*n* = 6) convened to provide an experience- and evidence-based guidance on rational drug use, patient education, and integrated and personalized nutrition care in both community and hospital pharmacy settings. A novel framework to guide community pharmacist-led consultations on supplementation is proposed. The four-step CARE (**C**ategorize, **A**ssess, **R**ecommend, **E**mpower) guide was developed to facilitate and optimize outcomes of pharmacist-led nutritional supplement consultation. Telehealth advancements in the form of digital health applications and personalized nutrigenomic DNA testing support Integrative Nutrition Care, and will further promote appropriate supplementation use to improve overall well-being in the community. Practical implementation of the CARE guide is necessary to ascertain its applicability for optimizing outcomes of pharmacist-led consultation and the recommendation of nutritional supplements.

## 1. The Role of Supplementation in Modern Society

Globally, there is a suboptimal average intake of foods such as whole grains, milk, fruits and vegetables [1], and an insufficient intake of key micronutrients such as calcium, iron, zinc, and vitamins A, B, and C [2]. Reflecting this global challenge, Malaysians today face challenges in achieving optimal nutrition that are common among developing, urbanized societies. Malaysia has one of the world’s poorest levels of work–life balance [3], with nine in ten Malaysian adults lacking a healthy and balanced diet [4]. The Malaysian Adult Nutrition survey conducted in 2014 found that the median intake of vitamin C and vitamin B1 was about half of the recommended nutrient intake, while that for calcium was less than half of the recommended level [5]. With an established link between nutrient insufficiency and development of diseases [1], suboptimal nutrition remains an obstacle to overall health promotion. These challenges become even more critical with the global trend of aging societies. By 2030, Malaysia is expected to become an aging nation where over 15% of its population will be older than 60 [6]. Such a projection highlights the rising importance of good nutrition to support healthy aging. 

Supplementation of vitamins and minerals has an important role in bridging observed nutritional gaps, and this is reflected by the steady increase in nutritional supplement use in Malaysia [6]. In Malaysia, the top reasons cited for the use of nutritional supplements were “general health and immunity”, “healthy aging and beauty”, and “bone and joint health” [6], indicating the emphasis that Malaysians place on healthy aging. In parallel, the Malaysian Dietary Supplement Association reported that the prevalence of vitamin and mineral supplementation appeared consistent (>20%) across various demographics, including rural and urban areas; females and males; main ethnic groups (Malay, Chinese, and Indian); and adults aged between 20 and 59 [6]. Today, easy accessibility to the internet offers consumers the convenience of buying nutritional supplements and seeking guidance on supplementation online. Inadequate guidance on supplementation, however, can propagate misconceptions and increase undue fears of side effects, resulting in low motivation and compliance to nutritional supplements. Pharmacists have a pivotal role in [7] overcoming misconceptions and other common barriers to appropriate supplementation. Our previous cross-sectional self-administrated survey-based study among pharmacists with at least 1 year working experience, demonstrated that the pharmacy curriculum does prepare pharmacists to understand complementary medicine. However, there is still a lack of competency trainings in dispensing supplements [8]. A survey conducted by Wong et al. on 453 community pharmacists in Malaysia demonstrated that only 66% of them had received complementary medicine education during their undergraduate studies [9]. Up to 75% of these pharmacists obtained the knowledge through self-directed training. Despite this, community pharmacists in Malaysia accept the professional responsibilities in supplementation consultancy [10]. Therefore, continuous competency trainings in supplementation consultancy will better position community pharmacists to guide consumers on supplement intake [10], as demonstrated by the fluoride supplementation for optimum dental care [11], folic acid supplementation during pre-conception planning [7], and calcium and vitamin D supplementation for bone health [12]. The authors summarized their experience and expertise on consumer interactions pertaining to appropriate supplementation, in Table 1.

Drawing on their collective experience, a panel of community pharmacists, pharmacy academia, and dietitians (*n* = 6) convened in a workshop to further propose a new framework to guide fellow community pharmacists in conducting systematic consultations on supplementation, to drive more effective and appropriate use of nutritional supplements within the community.

## 2. Facilitating Pharmacist-Led Consultation and Recommendation of Nutritional Supplements: A Four-Step CARE Guide

**Figure 1:**
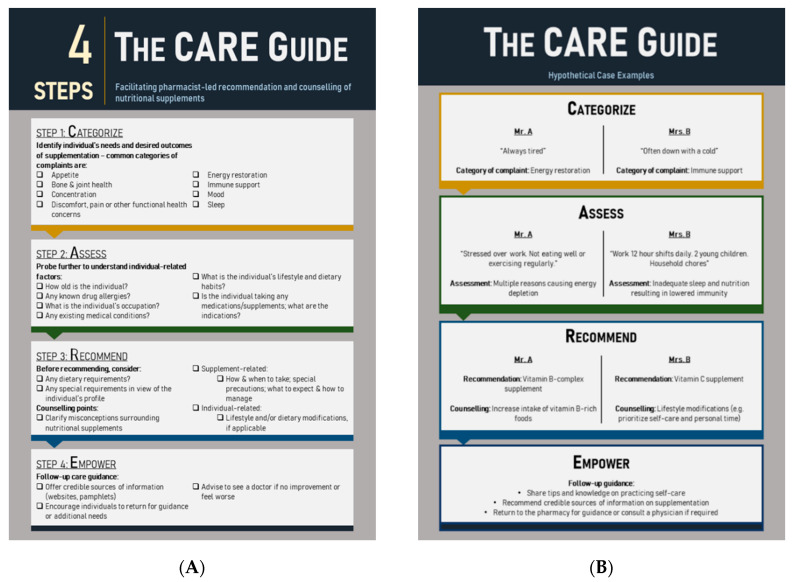
Guidance framework for pharmacist-led consultations on supplementation (**B**). The Categorize, Assess, Recommend, Empower (CARE) guide (**A**). Two hypothetical case examples illustrate how the CARE guide might be used in a real-world setting.

The consultation process between a community pharmacist and the consumer is the foundation of a longstanding relationship. Professionalism, approachability, empathy, and proactiveness are qualities of community pharmacists that are important in building this pharmacist–consumer relationship. Considering these attributes, the authors propose a four-step CARE (**C**ategorize, **A**ssess, **R**ecommend, **E**mpower) approach to facilitate and optimize the outcomes of community pharmacist-led nutritional supplement consultation (Figure 1A). The CARE model is similar to the widely adopted Pharmacist-Patient Care Process (PPCP) [13], which was developed by the United States Joint Commission of Pharmacy Practitioners and is now a requirement for PharmD accreditation program. PPCP was developed based on extensive evidence from pharmaceutical care and comprehensive medication therapy management, where pharmacists developed individualized patient-centered care plan in collaboration with other health care professionals and patients, family, and caregivers. The CARE model has a similar step-by-step patient-centric approach, with specific emphasis on nutrition care by pharmacists in the community setting.

Categorizing an individual’s needs allows pharmacists to rapidly define the consultation scope, a critical consideration in community pharmacy settings where consumer flow is especially fast-moving. Pharmacists should pay attention to any signs, behaviors, and verbalized wants or needs that may reveal the individual’s perceived reasons for seeking supplementation. For example, a hypothetical “Mr. A” who complains about tiredness may be categorized as someone looking for energy restoration options, while “Mrs. B” who is suffering from a cold can be categorized as someone looking for immune support (Figure 1B).

Asking questions to assess relevant factors for supplementation should follow immediately after categorization. Assess—the second step in the proposed CARE guide—involves asking social care questions relating to lifestyle, dietary habits, existing health conditions, occupation, and concurrent medications. This minimizes the risk of missing potential underlying health conditions in each individual. For example, effective probing reveals that Mr. A is stressed over work and has not been eating well or exercising often, thereby contributing to energy depletion. To take a step further, pharmacists should also probe deeper by asking Mr. A how long has he been experiencing this, and if there are any other stress factors that could contribute to his lack of energy, alongside a detailed medical and medication history. Meanwhile, Mrs. B is revealed to be a working mother who works long hours and also tends to household chores; her lack of sleep and inadequate nutrition thus result in lowered immunity (Figure 1B). Pharmacists should also proactively review any medication and/or supplement that the individual is consuming. Doing so will offer insights into any potential issues arising from drug–nutrient interactions, contraindications, or other factors.

Recommendation of nutritional supplements follows categorization and assessment. Recommendations must be accompanied by personalized advice derived from thorough understanding and consideration of the individual’s health and social care needs, which includes counselling points specific to the nutritional supplement (e.g., dose to take, when to take, compliance, and how to manage potential side effects) and the individual (e.g., accompanying dietary and lifestyle modifications and affordability). With a clear assessment of the individual’s lifestyle and preference, the pharmacist should be able to recommend suitable product formulations. For example, the effervescent tablet formulation can be recommended for individuals looking for easy and palatable supplement consumption. For Mr. A, the pharmacist may recommend vitamin B complex and advise him to increase his intake of vitamin-B-rich foods. For Mrs. B, the pharmacist may recommend vitamin C and suggest lifestyle modifications such as prioritizing self-care and personal time as a mother (Figure 1B). In this stage of the CARE guide, pharmacists can proactively counsel and debunk any misconceptions about supplementation to improve compliance. For example, pharmacists can inform consumers that urine discoloration is common when taking vitamin B; there is no need to be alarmed, as excess doses of vitamin B will not be absorbed by the body due to its water-soluble nature. Consumers can also be informed that vitamin C—also a water-soluble vitamin—is not associated with any adverse effects that were thought to be caused by excess vitamin C intake in healthy individuals [14].

Finally, community pharmacists are encouraged to conclude each consultation by empowering individuals to optimize the benefits of supplementation. For example, pharmacists could share tips and knowledge with individuals on how to practice self-care, and may refer consumers to credible sources of information (e.g., pamphlets, websites) or advise them to consult a physician if their condition or symptoms persist (Figure 1B). Individuals should also be encouraged to come back to the pharmacy for follow-up reviews.

## 3. Envisioning the Future: Integrative Nutrition CARE

Integrative Nutrition Care is envisioned by the authors as the provision of optimal nutrition care to consumers through a concerted effort between multidisciplinary healthcare practitioners (e.g., community pharmacists, dietitians, general practitioners, and specialists). The goal of Integrative Nutrition Care is to move toward person-centered care. Many community pharmacies are also gradually adopting a multidisciplinary care model, where dietitians partner pharmacists in consumer consultations. Dietitians are healthcare professionals who promote nutritional well-being and manage medical conditions through nutrition therapy. Several successful interventions were reported through the synergistic pharmacist–dietitian partnerships in this multidisciplinary healthcare ecosystem. In Australia, a rural community pharmacist partnered with a dietitian to initiate a healthy lifestyle program for young adults [15]. The program demonstrated a statistically significant clinical reduction in young adults’ body weight, waist circumference, healthy dietary habits, and physical activities. Another successful intervention was from a hypertension-focused program initiated by both a dietitian and a community pharmacist in the USA [16]. This intervention improved participants’ knowledge on hypertension and improved their medication adherence. Another partnership involving pharmacists, dietitians, and other healthcare professionals in an interdisciplinary diabetes team improved glucose control in a primary care rural setting [17]. These examples strongly suggest that when dietitians and pharmacists work in tandem, their distinct but complementary skillsets facilitate more comprehensive care for consumers, which encompasses nutrition care.

Genetic-related testing laboratories in Europe and the USA have already partnered with pharmacies in order to reach out to the public [18]. With current interest in healthy aging expected to rise in the future, consumers are increasingly empowered to take charge of their health. Personalized supplementation has become increasingly popular and possible with nutrigenomic DNA testing, which uses genetic data to provide individuals with customized information on their nutrient and dietary needs. Nutrigenomic DNA testing may provide insights on how dietary substances may react to a specific sequence of one’s genome; be a possible risk factor for a known disorder; affect the balance between healthy and disorder; induce onset, progress, or worsening of a disorder; and be used to prevent, mitigate, or prevent a disorder [19]. Today, consumers are ready to employ nutrigenomics regardless of potential risks because they believe nutrigenomics analysis can lead to earlier diagnoses as well as disease prevention through healthier dietary habits [20,21]. Therefore, nutrigenomic DNA testing should be accompanied by consultations with healthcare professionals such as pharmacists, dietitians, or physicians, for accurate interpretation of results and professional advice on appropriate next steps. A study in Greece suggested that 80.5% of healthcare professionals are keen to recommend that the public undergo nutrigenomic analysis to tailor their diets according to their personal genetic profile [22]. By offering personalized dietary advice [23], nutrigenomics can empower consumers to have a wider interest in their dietary habits, thereby triggering long-lasting behavior change in self-care and achieving target interventions faster. With this technological advancement in nutrition care, a wholistic regulatory framework is required to adequately address all the ethical, legal, and social concerns that may arise from nutrigenomics [23]. Pharmacists will play a critical role here to guide and support consumers in the use and interpretation of nutrigenomic DNA testing.

Telehealth advancements in the form of digital health applications also have the potential to support pharmacist-centric Integrative Nutrition Care [24]. Digital health coaching applications offer consumers convenience and accessibility to holistic consultation services with multidisciplinary healthcare practitioners (e.g., pharmacists, dietitians, health coaches, physicians, and psychologists) based on their specific needs [24,25].

The authors acknowledge two main limitations of the proposals put forth in this article. While advancements in nutrigenomic testing and telehealth have the potential to promote a holistic and robust healthcare ecosystem [25], unequal access to technology currently may limit its uptake and application in an Integrative Nutrition Care model. Next, the applicability of the proposed CARE guide needs to be further ascertained and optimized in actual community pharmacy practice. Future possibilities include piloting the CARE guide in community pharmacies, where real-world feedback from consumers and pharmacists may aid in the refinement of the CARE guide for continued use as part of an Integrative Nutrition Care.

## 4. Conclusions

Healthcare professionals, such as pharmacists, are well-positioned within community pharmacies to provide consumers with guidance and counselling on nutritional supplement use. The proposed four-step CARE (**C**ategorize, **A**ssess, **R**ecommend, **E**mpower) guide serves to optimize pharmacist-led supplement consultations. Integrative Nutrition Care can be achieved through strong partnerships between multidisciplinary healthcare practitioners and enhanced by telehealth advancements. Practical implementation of the CARE guide in community pharmacies is necessary to assess its applicability for optimizing outcomes of pharmacist-led consultation and the recommendation of nutritional supplements.

## Figures and Tables

**Table 1:** Identified consumer barriers to appropriate supplementation and the possible roles of community pharmacists.

**FIRST APPROACH**		
	**Identified Consumer Barriers**	**Role of Community Pharmacists**
Reasons for supplementation	Lack of clarity on their reasons for supplementation	Understand consumers’ reasons for supplementation, thereby helping consumers articulate their needs
Selection of nutritional supplement(s)	Lack of knowledge on the appropriate selection of nutritional supplements	Recommend nutritional supplements that are best suited to the consumer’s profile and needs
**CONSULTATION**		
	**Identified Consumer Barriers**	**Role of Community Pharmacists**
Appropriate use of the recommended nutritional supplement	Lack of precise information on how to correctly consume nutritional supplements, thereby affecting compliance and outcome of supplementation	Equip consumers with essential information on the recommended nutritional supplement and directions for use—e.g., indication, dosage, and storage
Supplement-specific considerations	Lack of credible information may propagate misconceptions and cause undue fears of side effects that may hinder compliance.	Counsel consumers on what to expect when taking the recommended nutritional supplement, and how to manage potential side effects Clarify and debunk any misconceptions regarding side effects
**CONTINUATION OF CARE**		
	**Identified Consumer Barriers**	**Role of Community Pharmacists**
Motivation and compliance during recommended supplementation period	Lack of motivation to comply with nutritional supplement recommendations over time	Encourage and empower consumers by helping them see how supplementation can help them meet their articulated needs
Continued supplementation guidance	Requirements and use of nutritional supplements that may change over time	Foster a strong pharmacist-consumer relationship to allow continued assessment of consumers’ needs and to encourage compliance with the recommended supplementation
Support groups	Lack of understanding on how to self-care	Educate, refer, and connect consumers to relevant support groups, where they can interact with like-minded individuals

## Abbreviations

CARE	Categorize: Assess: Recommend, Empower
DNA	Deoxyribonucleic Acid
PharmD	Doctor of Pharmacy
PPCP	Pharmacist-Patient Care Process
USA	United States of America
